# Association Between Antenatal Antimicrobial Therapy and Autism Spectrum Disorder—A Nested Case-Control Study

**DOI:** 10.3389/fpsyt.2021.771232

**Published:** 2021-11-19

**Authors:** Nitzan Abelson, Gal Meiri, Shirley Solomon, Hagit Flusser, Analya Michaelovski, Ilan Dinstein, Idan Menashe

**Affiliations:** ^1^Joyce & Irving Goldman Medical School, Faculty of Health Sciences, Ben-Gurion University of the Negev, and Soroka University Medical Center, Beer-Sheva, Israel; ^2^Pre-School Psychiatry Unit, Soroka University Medical Center, Beer-Sheva, Israel; ^3^National Autism Research Center of Israel, Beer-Sheva, Israel; ^4^Child Development Center, Soroka University Medical Center, Beer-Sheva, Israel; ^5^Psychology Department, and Cognitive and Brain Sciences Department, Ben-Gurion University of the Negev, Beer-Sheva, Israel; ^6^Zlotowski Center for Neuroscience, Ben-Gurion University of the Negev, Beer-Sheva, Israel; ^7^Public Health Department, Faculty of Health Sciences, Ben-Gurion University of the Negev, Beer-Sheva, Israel

**Keywords:** Autism spectrum disorder (ASD), prenatal, antimicrobial drugs, public health, ethnic disparities

## Abstract

**Background:** Multiple prenatal factors have been associated with autism spectrum disorder (ASD) risk. However, current data about the association between antimicrobial use during pregnancy and ASD is limited.

**Methods:** A nested matched case-control study of children with ASD (cases), and children without ASD or other psychiatric or genetic disorders (controls). We compared the use of antimicrobial therapy during the 3 months before conception or during pregnancy between mothers of cases and controls and used multivariate conditional logistic regression models to assess the independent association between maternal use of antimicrobials during pregnancy and the risk of ASD in their offspring.

**Results:** More than half of the mothers in the study (54.1%) used antimicrobial drugs during the 3 months before conception or during pregnancy. Rates of antimicrobial use were lower for mothers of children with ASD compared to mothers of controls (49.0 vs. 55.1%, respectively; *p* = 0.02), especially during the third trimester of pregnancy (18.8 vs. 22.9%, respectively; *p* = 0.03), and for the use of penicillins (15.7 vs. 19.7%, respectively; *p* = 0.06). These case–control differences suggest that antimicrobial administration during pregnancy was associated with a reduced risk of ASD in the offspring (aOR = 0.75, 95% CI = 0.61–0.92). Interestingly, this association was seen only among Jewish but not for the Bedouin mothers (aOR = 0.62, 95% CI = 0.48–0.79 and aOR = 1.21, 95% CI = 0.82–1.79).

**Conclusions:** The reduced risk of ASD associated with prenatal antimicrobials use only in the Jewish population suggest the involvement of other ethnic differences in healthcare services utilization in this association.

## Introduction

Autism spectrum disorder (ASD) is a neurodevelopmental condition, characterized by deficits in social communication and interaction and accompanied by the presence of restrictive, repetitive behaviors, interests, or activities (1, 2). ASD is a major public health issue, with healthcare expenditure on children with ASD being nine times higher than that for children with normal development, and three times higher than that for children with intellectual disabilities (3). Of particular concern is the remarkable rise in the prevalence of ASD in the past few decades; for example, in the USA, ASD rates rose from approximately 1 in 150 children at the beginning of this century (4) to 1 in 54 children under the age of eight by 2016 (5). It is likely that the dramatic increase in ASD prevalence stems from a better awareness of the condition among physicians and parents, accompanied by changes in ASD diagnostic criteria. However, the contribution to ASD prevalence of new risk factors or changes in lifestyle habits cannot be excluded (3, 6–8).

It is commonly accepted that the prenatal period plays a significant role in ASD susceptibility, and a wide range of prenatal risk factors have been associated with ASD (6–13). One of these factors is maternal infection during pregnancy, with some studies reporting a stronger association with infections requiring hospitalization and with multiple infections during pregnancy (7, 14–18). Other studies, including some on animal models, have suggested that maternal immune activation rather than the infection itself is the cause of the elevated risk of ASD (7, 19, 20). In parallel, an association between maternal fever and ASD has been demonstrated, with a stronger association with ASD for a longer duration of fever (21, 22). In this regard, the use anti-pyretic treatment during pregnancy has been suggested to have a protective effect on ASD risk (6, 7, 13, 23). Consequently, it is not clear whether the reported association between prenatal infection and ASD is due to exposure to an infectious organism, the immune response associated with the resulting infection, the corresponding medical treatment, or a combination of these factors (6, 7).

The treatment of an infection during pregnancy usually includes treatment with antimicrobials, which account for ~80% of all medications prescribed to pregnant women (24). It is estimated that 19–49% of pregnant women receive antimicrobial agents during pregnancy and that this percentage will continue to increase (24–29). The use of antimicrobial therapy during pregnancy has been associated with various unwanted outcomes in the offspring, including cerebral palsy, epilepsy, immune system alterations, childhood asthma, changes in gut microbiota, obesity, and functional impairments (24). The effect of antimicrobial use during pregnancy on ASD risk is not yet clear: while some studies have reported that the use of antimicrobials during pregnancy is associated with an increased risk of ASD (17, 21, 30–34), other studies did not find such association (35). Therefore, the objective of this study was to investigate the association between the use of antimicrobials during pregnancy and the risk of ASD in the offspring.

## Methods

### Study Design

We conducted a nested case-control study of children who were born at the Soroka University Medical Center (SUMC), Beer-Sheva, between the years 2008 and 2016 and who were members of Clalit, the largest Health Maintenance Organization (HMO) in Israel. SUMC is the only tertiary hospital in southern Israel and is associated with the Clalit HMO. Cases were defined as children who were diagnosed with ASD at SUMC and who were enrolled in the database of the National Autism Research Center of Israel (NARCI), which is a repository of comprehensive medical data pertaining to those children and their families (36, 37). Controls were children with no diagnosis of ASD, or other psychiatric or genetic disorders, who were randomly sampled from the computerized database of the SUMC and were individually matched to the case group at a ratio of 5:1 according to age, sex, and ethnic origin (Jewish or Bedouin). Exclusion criteria included incomplete maternal records and children with other neurodevelopmental or psychiatric disorders.

### Data Acquisition

Medical and sociodemographic data for both cases and controls were obtained from the SUMC computerized database, which contains electronic records of all medical treatments given at the hospital as well as medical data from the outpatient clinics of the Clalit HMO. For this study, we extracted complete pregnancy and birth records for all children in the study. In addition, we obtained basic sociodemographic data for the mothers as well as data regarding chronic conditions and regularly prescribed medications that were recorded prior to childbirth. Data about antimicrobial use prior to (up to 3 months before conception) and during the pregnancy included antimicrobial type, which was encoded in the database according to the Anatomical Therapeutic Chemical (ATC) Classification. The method of administration was classified to topical use (cream or ointment), mucosal (cream, ointment, or a suppository for use orally, rectally, or vaginally) or systemic (oral, intramuscular, or intravenous). The trimester in which the drug was given was calculated using the date of the prescription and the week of the child's delivery. Unfortunately, the medical indication associated with each drug prescription was not available in SUMC database, since physicians are not required to document this information in the computerized system. Antimicrobial treatments with a prevalence of <1% were aggregated with other variables of the same category or were removed from the analyses. All antimicrobial treatments included in this study are listed in Supplementary Table 1.

### Statistical Analysis

We used standard univariate statistics to compare sociodemographic and medical variables between cases and controls. Variables with statistically significant case-control differences (*p* < 0.05) were included in multivariable conditional logistic regression models that were used to assess the association between the type of antimicrobial therapy with the risk of ASD. We also performed a sensitivity analysis by testing the association between prenatal antimicrobial and ASD in the offspring separately for the Jewish and Bedouin mothers, since these two ethnic groups are known to differ significantly in their access to medical care and their ASD prevalence (38). We used the Breslow-Day test (39) to assess the homogeneity of the odds ratios (ORs) between these two ethnic groups. The statistical analysis was performed using the IBM SPSS Statistics software, version 23.0, and R studio, version 1.1.456 (R Foundation for Statistical Computing version 3.4.4). This study was approved by the SUMC ethics committee (SOR 222-14).

## Results

Overall, 451 cases and 2,255 controls were included in this study. Of these 2,706 children, 81% were males and 71% were of Jewish origin. Table 1 presents the basic demographic and clinical characteristics of mothers of cases and controls. Mothers of cases were of higher socioeconomic status than mothers of controls (median socioeconomic level of 8 vs. 6, *p* ≤ 0.01) and had higher rates of obesity (10.0 vs. 5.9%; *p* ≤ 0.01), epilepsy (2.2 vs. 0.6%; *p* ≤ 0.01), and hypertension (5.3 vs. 3.2%; *p* = 0.02). Mothers of cases also had statistically significantly higher rates of certain chronic medication prescriptions, specifically, inhaled adrenergic (4.2% in ASD vs. 2.4% in non-ASD; *p* = 0.04) and anti-hyperglycemic (2% in ASD vs. 0.7% in non-ASD; *p* ≤ 0.01) medications compared to mothers of controls.

**Table 1:** Demographic and clinical characteristics of mothers of children with ASDs and matched non-ASDs controls^*^.

**Variable**	ASD^**^	Non-ASD^**^	*P*-value^**^
	***N* = 451**	***N* = 2,255**	
**Demographic characteristics**			
Age [mean years (±SD)]	29.48 (± 5.9)	29.02 (± 5.47)	0.11
Socioeconomic status [median (IQR)]	**8 (3–11)**	**6 (0–10)**	**<0.01**
**Chronic medical conditions**			
Obesity [*N* (%)]	**45 (10.0%)**	**134 (5.9%)**	**<0.01**
Any psychiatric [*N* (%)]	12 (2.7%)	39 (1.7%)	0.20
Depression	8 (1.8%)	28 (1.2%)	0.37
Other psychiatric	5 (1.1%)	9 (0.4%)	0.08
Epilepsy [*N* (%)]	**10 (2.2%)**	**13 (0.6%)**	**<0.01**
Asthma [*N* (%)]	38 (8.4%)	161 (7.1%)	0.41
Atopic dermatitis [*N* (%)]	15 (3.3%)	94 (4.2%)	0.49
Diabetes mellitus [*N* (%)]	23 (5.1%)	75 (3.3%)	0.12
Any hypertension [*N* (%)]	**24 (5.3%)**	**73 (3.2%)**	**0.02**
Thyroid [*N* (%)]	26 (5.8%)	146 (6.5%)	0.60
Hyperlipidemia [*N* (%)]	5 (1.1%)	16 (0.7%)	0.37
Rheumatic [*N* (%)]	13 (2.9%)	52 (2.3%)	0.41
**Chronic medications**			
Antiepileptic [*N* (%)]	7 (1.6%)	24 (1.1%)	0.42
Psychiatric any [*N* (%)]	11 (2.4%)	63 (2.8%)	0.63
SSRI	8 (1.8%)	30 (1.3%)	0.48
Asthmatic [*N* (%)]	21 (4.7%)	86 (3.8%)	0.48
Inhaled adrenergic [*N* (%)]	**19 (4.2%)**	**55 (2.4%)**	**0.04**
Anti-hyperglycemic [*N* (%)]	**9 (2.0%)**	**15 (0.7%)**	**<0.01**
Antihypertensive [*N* (%)]	8 (1.7%)	18 (0.8%)	0.06
Antacids [*N* (%)]	46 (10.2%)	193 (8.6%)	0.30
Glucocorticosteroids [*N* (%)]	14 (3.1%)	61 (2.7%)	0.75

* *Cases and controls were matched according to sex, date of birth, and ethnic origin*.

** *Bold text indicates significant case-control differences at α < 0.05*.

Rates of antimicrobial prescriptions during the preconception and pregnancy periods are shown in Table 2. More than half of the women in the study (54.1%) were prescribed an antimicrobial treatment during the preconception or pregnancy periods. Mothers of children with ASD had lower rates of antimicrobial drug prescriptions prior to or during pregnancy than mothers of non-ASD children (49.0 vs. 55.1%, respectively; *p* = 0.02), with the largest difference between these groups seen in the third trimester (18.8 vs. 23.7%; *p* = 0.03). The most prevalent antimicrobial treatments were beta lactam drugs (penicillins and cephalosporins; *N* = 983), followed by antifungal medications (*N* = 644), with these two types of drugs accounting for over 75% of all administered antimicrobial agents. Notably, there was a marginally insignificant difference in the prescription of penicillins between the groups, with mothers of cases having lower rates of penicillin prescriptions during pregnancy than mothers of controls (15.7 vs. 19.7%; *p* = 0.06). In addition, mothers of cases had significantly higher rates of systemic antifungal drugs prescriptions than mothers of controls (1.3 vs. 0.4%; *p* = 0.03). There were no other significant differences in prescription rates of specific antimicrobial drugs between the study groups.

**Table 2:** Antimicrobials taken by mothers of children with ASDs and matched non-ASDs controls^*^.

**Variable**		**All**	**ASD**	**Non-ASD**	***P*-value**
		***N* = 2,706**	***N* = 451**	***N* = 2,255**	
Any antimicrobial [*N* (%)]		**1,463 (54.1%)**	**221 (49.0%)**	**1,242 (55.1%)**	**0.02**
Antimicrobial period [*N* (%)]	3 Months before conception	700 (25.9%)	107 (23.7%)	593 (26.3%)	0.28
	Trimester 1	762 (28.2%)	118 (26.2%)	644 (28.6%)	0.33
	Trimester 2	760 (28.1%)	123 (27.3%)	637 (28.2%)	0.72
	Trimester 3	**619 (22.9%)**	**85 (18.8%)**	**534 (23.7%)**	**0.03**
Antimicrobial type [*N* (%)]	Any mucosal	423 (15.6%)	71 (15.7%)	352 (15.6%)	1
	Any topical	674 (24.9%)	99 (22%)	575 (25.5%)	0.13
	Any systemic	1,072 (39.6%)	163 (36.1%)	909 (40.3%)	0.11
	Systemic Beta-lactam	983 (36.3%)	150 (33.3%)	833 (36.9%)	0.15
	Penicillin	514 (19.0%)	71 (15.7%)	443 (19.7%)	0.06
	Cephalosporin	647 (23.9%)	101 (22.4%)	546 (24.2%)	0.44
	Macrolide	51 (1.9%)	4 (0.9%)	47 (2.1%)	0.13
	Antifungal	644 (23.8%)	103 (22.8%)	541 (24%)	0.64
	Mucosal	398 (14.7%)	67 (14.9%)	331 (14.7%)	0.98
	Topical	450 (16.6%)	65 (14.4%)	385 (17.1%)	0.19
	Systemic	**15 (0.6%)**	**6 (1.3%)**	**9 (0.4%)**	**0.03**
	Aminoglycoside topical	94 (3.5%)	12 (2.7%)	82 (3.6%)	0.37
	Antiviral topical	50 (1.8%)	7 (1.6%)	43 (1.9%)	0.75
	Chloramphenicol topical	103 (3.8%)	16 (3.6%)	87 (3.9%)	0.86
	Other topical	46 (1.7%)	10 (2.2%)	36 (1.6%)	0.46

* *Cases and controls were matched according to sex, date of birth, and ethnic origin*.

*Bold text indicates significant case-control differences at α < 0.05*.

We then used multivariate conditional logistic regression models to assess the independent association of antimicrobial agents with ASD risk. Each such model included a particular antimicrobial drug together with demographic, socioeconomic, and clinical variables associated with ASD, as defined in Table 1. The results of these multivariate models are presented in Table 3. Overall, prescription of any antimicrobial treatment during pregnancy was associated with a protective effect on ASD risk (aOR = 0.75; 95%CI = 0.61–0.92). This association was driven mainly by drugs used in the third trimester of pregnancy (aOR = 0.73; 95%CI = 0.57–0.95) and by systemically administered medications (aOR = 0.79; 95%CI = 0.64–0.98). Notably, while penicillins and macrolides were significantly associated with a protective effect on the risk of ASD (aOR = 0.71; 95%CI = 0.54–0.94 and aOR = 0.34; 95%CI = 0.12–0.97, respectively), systemically administered antifungal medications during pregnancy were significantly associated with an increased risk of ASD in the offspring (aOR = 3.85; 95%CI = 1.34–11.01). However, the number of pregnant women treated with systemic antifungals in this sample was low (*N* = 15).

**Table 3:** Crude and adjusted ORs for ASD of different types of antimicrobial agents in a multivariate model including background characteristics.

**Antimicrobial agent**	**Crude OR (95% CI)**	**aOR** **^#^ (95% CI)**
Any antimicrobial (*N* = 1,463)	0.79 (0.65–0.97)^*^	0.75 (0.61–0.92)^**^
3 months pre-conception (*N* = 710)	0.88 (0.7–1.12)	0.8 (0.63–1.02)
Trimester 1 (*N* = 771)	0.89 (0.71–1.12)	0.86 (0.68–1.09)
Trimester 2 (*N* = 767)	0.97 (0.77–1.22)	0.93 (0.74–1.18)
Trimester 3 (*N* = 621)	0.75 (0.58–0.97)^*^	0.73 (0.57–0.95)^*^
Any systemic antimicrobial (*n* = 1,072)	1.03 (0.78–1.37)	0.79 (0.64–0.98)^*^
Any mucosal antimicrobial (*n* = 423)	0.83 (0.65–1.06)	1.03 (0.78–1.38)
Any topical antimicrobial (*n* = 674)	0.85 (0.69–1.04)	0.8 (0.62–1.02)
Systemic Beta-lactam (*n* = 983)	0.86 (0.69–1.06)	0.8 (0.65–1.02)
Penicillin (*n* = 514)	0.78 (0.59–1.02)	0.71 (0.54–0.94)^*^
Cephalosporin (*n* = 647)	0.91 (0.72–1.16)	0.86 (0.67–1.10)
Macrolide (*N* = 51)	0.41 (0.15–1.16)	0.34 (0.12–0.97)^*^
Antifungal (*N* = 644)	0.95 (0.74–1.2)	0.93 (0.73–1.19)
Mucosal (*n* = 398)	1.04 (0.78–1.38)	1.03 (0.77–1.38)
Topical (*n* = 450)	0.83 (0.62–1.1)	0.8 (0.6–1.07)
Systemic (*n* = 15)	3.28 (1.17–9.23)	3.83 (1.34–11.01)^*^
Aminoglycoside topical (*n* = 94)	0.73 (0.4–1.35)	0.72 (0.39–1.34)
Antiviral topical (*n* = 50)	0.83 (0.37–1.85)	0.7 (0.31–1.59)
Chloramphenicol topical (*n* = 103)	0.93 (0.54–1.6)	0.98 (0.57–1.71)
Other topical (*n* = 46)	1.39 (0.69–2.8)	1.29 (0.63–2.68)

* *Significant at α = 0.05*.

** *Significant at α = 0.01*.

# *Adjusted ORs to the following variables: age, low socioeconomic status, obesity, epilepsy, hypertension, anti-hyperglycemic medication, and inhaled adrenergic medication*.

Finally, we performed a sensitivity analysis exploring the association between antimicrobial drug prescriptions during pregnancy and risk of ASD in the offspring of Jewish and Bedouin mothers, two ethnic groups that differ in their sociodemographic and clinical characteristics (Supplementary Table 2). The results of this analysis are presented in Table 4. Interestingly, a significant difference was seen in the risk of ASD associated with antimicrobial drug prescription during pregnancy in these two populations (Breslow-Day *p*-value < 0.01). Specifically, any antimicrobial drug prescription during pregnancy was associated with a reduced risk of ASD in the offspring of Jewish mothers (aOR = 0.61; 95%CI = 0.48–0.79) but not of Bedouin mothers (aOR = 1.18; 95%CI = 0.79–1.75). This ethnic difference in ASD risk was mainly driven by any systemic antimicrobial drugs (aOR_Jewish_ = 0.62; 95%CI = 0.47–0.81 and aOR_Bedouin_ = 1.31; 95%CI = 0.89–1.92, respectively) and primarily by penicillins (aOR_Jewish_ = 0.55; 95%CI = 0.38–0.78 and aOR_Bedouin_ = 1.18; 95%CI = 0.73–1.90, respectively).

**Table 4:** Adjusted ORs for ASD of different types of antimicrobial agents in a multivariate model including background characteristics, stratified by ethnicity.

**Antimicrobial agent**	**Jews aOR**^#^** (95% CI)**	**Bedouins aOR**^#^** (95% CI)**	**Breslow day**
	**(*N* = 1,914)**	**(*N* = 792)**	***P*-value**
**Any antimicrobial (*****N*** **=** **1,463)**	0.61 (0.48–0.79)^***^	**1.18 (0.79–1.75)**	** <0.01**
3 months pre-conception (*N* = 710)	0.86 (0.64–1.14)	0.7 (0.43–1.12)	0.59
Trimester 1 (*N* = 771)	0.76 (0.57–1.01)	1.03 (0.67–1.58)	0.22
Trimester 2 ((*N* = 767)	0.87 (0.66–1.15)	1.08 (0.70–1.67)	0.17
Trimester 3 (*N* = 621)	0.66 (0.48–0.91)^**^	1 (0.63–1.59)	0.13
**Any systemic antimicrobial (*****n*** **= 1,072)**	0.62 (0.47–0.8)^***^	**1.31 (0.89–1.92)**	** <0.01**
Any mucosal antimicrobial (*n* = 423)	1 (0.72–1.40)	1.21 (0.68–2.13)	0.52
Any topical antimicrobial (*n* = 674)	0.77 (0.58–1.03)	0.78 (0.47–1.3)	0.79
**Systemic Beta–lactam (*****N*** **= 983)**	0.65 (0.49–0.85)^**^	**1.23 (0.83–1.81)**	**0.01**
Penicillin (*N* = 514)	0.55 (0.38–0.78)^***^	1.18 (0.73–1.90)	0.02
Cephalosporin ((*N* = 647)	0.75 (0.55–1.03)	1.05 (0.69–1.62)	0.20
Macrolide (*N* = 51)	0.2 (0.05–0.86)^*^	1.07 (0.23–5.06)	0.15
Antifungal (*N* = 644)	0.92 (0.69–1.22)	1.04 (0.63–1.71)	0.63
Mucosal (*N* = 398)	0.97 (0.69–1.37)	1.31 (0.74–2.32)	0.36
Topical (*N* = 450)	0.8 (0.58–1.12)	0.79 (0.42–1.47)	0.99
Systemic (*N* = 15)	4 (1.07–14.94)^*^	3.48 (0.56–21.47)	0.99
Aminoglycoside topical (*n* = 94)	0.54 (0.24–1.21)	1.25 (0.46–3.41)	0.23
Antiviral topical (*n* = 50)	0.59 (0.22–1.55)	0.49 (0.07–3.39)	0.75
Chloramphenicol topical (*n* = 103)	0.89 (0.46–1.74)	1.14 (0.39–3.32)	0.40
Other topical (*n* = 46)	1.7 (0.79–3.65)	#N/A	0.08

* *Significant at α = 0.05*.

** *Significant at α = 0.01*.

*** *Significant at α = 0.001*.

# *Adjusted ORs to the following variables: age, low socioeconomic status, obesity, epilepsy, hypertension, anti-hyperglycemic medication, and inhaled adrenergic medication*.

## Discussion

The results of this study suggest that the administration of antimicrobial drugs during gestation is associated with a reduced risk of ASD in the offspring of Jewish mothers living in southern Israel. Furthermore, we show that this association is driven primarily by the administration of antimicrobials, particularly penicillins, during the third trimester of pregnancy. To the best of our knowledge, no other study has documented such a trend. Other studies that explored the association between antimicrobial use during pregnancy and ASD risk reported a positive or null association between these variables (16, 21, 40–42). The discrepancy between these studies and our results of a lower risk of ASD associated with prenatal use of antimicrobials could have several biological and/or clinical explanations, as described below.

Several hypotheses have been proposed regarding the association between antimicrobial therapy during pregnancy and risk of ASD. One of the main hypotheses, which may be relevant to this study, is related to the effect of antimicrobial treatment on the composition of the gut microbiota of the mother and consequently on that of her fetus. Alterations in the gut microbial flora may alter the functioning of the gut–brain axis in the developing fetus and lead to abnormalities in brain developments associated with ASD (6, 24, 31, 35, 43). Generally, alterations in the gut microbiota have been shown to be associated with an increased risk of ASD (43–45). Nevertheless, it is also possible that antimicrobials may confer a protective effect (like that observed in our study) by reducing the effect of harmful microbial species in the gut of either the fetus or the mother. In addition, antimicrobial administration during pregnancy may weaken the activation of the maternal immune system, which has been suggested as a factor causing an elevated risk of ASD (7, 19, 20). Another possible explanation is that microbial treatment shortens the illness of the mother and, therefore, reduces the negative effects of maternal fever on the risk of ASD (13, 19, 22, 23). Lastly, antimicrobial therapy during pregnancy may convey a protective effect through the treatment of secondary “silent” [asymptomatic (46)] infections that may influence the neurodevelopment of the fetus. These theories are supported by the protective effect of systemically administered antimicrobials (i.e., penicillins and macrolides) that target a broad range of bacteria.

The observation that a protective effect of prenatal antimicrobial intake on ASD risk was shown only for the Jewish mothers in this study is most probably related to cultural and/or environmental differences between Jewish and Bedouin people living in Israel. In this context, the prescription of antimicrobials may be regarded as a proxy for the utilization of healthcare services. The differences in the protective effect of antimicrobial therapy on ASD risk between the Bedouin and Jewish populations in this study may simply be related to differences in the quantity and/or quality of prenatal medical care between these two ethnic groups (47, 48). This association was observed mainly in the third trimester, concurrent with the requirement for more frequent prenatal visits during this trimester. An additional factor that must be taken into consideration is the practice of prescribing antimicrobial therapy prior to confirmation of a viral or bacterial etiology of the patient's illness. This over-prescription of antimicrobials, often driven by patients themselves seeking prompt medical care, rather than adopting a wait-and-see approach, may confound the relationship between antimicrobial therapy and risk of ASD in those mothers who seek increased medical care, particularly since those mothers may also be more likely to engage in other protective behaviors that, in turn, would positively affect the neurodevelopmental outcomes in their offspring. It is important to note that the protective effects, or associated protective effects, of antimicrobial use were not found in other studies, which may have studied different populations with similar utilizations of health services.

Notably, in contrast to all the other results in this study, systemic antifungal medications were associated with an increased risk of ASD. While this association may be due to the severity of the infection rather than to the iatrogenic effects of the prescribed antimicrobials (49, 50), this finding should be regarded with caution since only 15 women (0.6%) in our sample were prescribed this medication.

Among the strengths of this study is its design, namely, data was collected through the use of a clinical database rather than through retrospective questionnaires, thereby greatly increasing the internal validity of the data. In addition—as facilitated by the NARCI database—our sample size was relatively large compared to other studies on children with ASD, and we were able to include many known risk factors within the analyses. Lastly, in an attempt to mitigate potential limitations, clinical data on antimicrobial prescriptions were acquired from many different types of medical services via the SUMC database, including general practitioner visits, specialist clinics, emergency department visits, and hospitalization summaries.

Nevertheless, our findings should be considered in the context of the following study limitations. Although large, the sample size was not sufficiently large for analyses to include more than a few subcategories of antimicrobial agents. Secondly, the data about prenatal antimicrobial administration did not include information about the dosage, indicated illness for the prescription nor to the compliance with these prescriptions. Differences in prescription compliance between mothers of ASD and non-ASD offspring could have affected our results, but there are no indications for us to believe that such differences do indeed exist. Thirdly, we did not have data about the medical indications for the antimicrobial prescriptions, which limited further analyses on this equally important factor. Lastly, the study was conducted on data from mothers living in a specific geographical location and enrolled in a single HMO. Therefore, generalization of the study findings to other populations is limited. These limitations call for further research, which may help to better understand the association between antimicrobial use and ASD risk. Nonetheless, this study did reveal the important finding that prescription of antimicrobials, in the 3 months before conception and during pregnancy, do not convey any added risk of ASD in offspring.

## Conclusions

The results of our study shed some light on the possible positive effects of shared factors associated with antimicrobial treatment, such as high utilization of healthcare services during pregnancy, on the risk of ASD in the offspring of the treated mothers. Follow-up studies are required to establish the true nature of this association, as it may have significant clinical and scientific implications.

## Data Availability Statement

The raw data supporting the conclusions of this article will be made available by the authors, without undue reservation.

## Ethics Statement

The protocol of this study was reviewed and approved by the Ethic Committee of Soroka University Medical Center. Written informed consent from the participants' legal guardian/next of kin was not required to participate in this study in accordance with the national legislation and the institutional requirements.

## Author Contributions

NA conducted the data analysis. NA and SS drafted the manuscript. GM, HF, AM, and ID assisted in data collection and interpretation. NA and IM conceptualized the manuscript. IM coordinated the work and supervised the data analysis. All authors are responsible for the reported research and have reviewed and approved the final manuscript as submitted.

## Funding

This study was partially funded by a grant from the Israeli Science Foundation (Grant No. 527/15).

## Conflict of Interest

The authors declare that the research was conducted in the absence of any commercial or financial relationships that could be construed as a potential conflict of interest.

## Publisher's Note

All claims expressed in this article are solely those of the authors and do not necessarily represent those of their affiliated organizations, or those of the publisher, the editors and the reviewers. Any product that may be evaluated in this article, or claim that may be made by its manufacturer, is not guaranteed or endorsed by the publisher.
