# Supplementary material for: Association Between Antenatal Antimicrobial Therapy and Autism Spectrum Disorder—A Nested Case-Control Study
Source: Front Psychiatry. 2021 Nov 19;12:771232. doi: 10.3389/fpsyt.2021.771232 (PMC8639519; doi:10.3389/fpsyt.2021.771232)
Supplement: Supplementary file 1 [file Table_1.DOCX]

**Table S1:** ATC codes and free text of antimicrobial treatments included in this study in the raw database

| **Variable** | **Variable Meaning** | **Raw Database ATC Codes** |
| --- | --- | --- |
| AB_MYC_Mucosal | Antifungal mucosal | G01AF02, G01AF04, G01AF05 |
| AB_MYC_Topical | Antifungal topical | D01AC01, D01AC02, D01AC08, D01AC10, D01AC21, D01AC22, D01AC23, D01AC24, D01AC25, D01AC52, D01AC60, D01AC63,  D01AE04, D01AE14, D01AE15, D01AE18, D01AE50 |
| AB_MYC_Systemic | Antifungal systemic | D01BA02, J02AB02J02AC01, J02AC02, A01AB09 |
| AB_AG_Mucosal | Aminoglycoside mucosal | J01GB01, S01AA11, S01AA12 |
| AB_AG_Topical | Aminoglycoside topical | D06AX07, D07CA03, D07CC01, S01CA01,  S02CA06 |
| AB_AG_Systemic | Aminoglycoside systemic | J01GB03, J01GA01 |
| AB_Antiviral_Topical | Antiviral topical | D06BB02, D06BB03, D06BB04, D06BB06,  S01AD03 |
| AB_Antiviral_Systemic | Antiviral systemic | J05AB01, J05AF05, J05AH02 |
| AB_SULFA_Topical | Sulfonamide topical | D06BA01, S01AB04, S01CA02 |
| AB_SULFA_Systemic | Sulfonamide systemic | J01EE01 |
| AB_TTCN | Tetracycline | J01AA02, J01AA07, J01AA08, S01AA30 |
| AB_MCD | Macrolide | D10AF02, D10AF52, J01FA01, J01FA06,  J01FA09, J01FA10 |
| AB_Imidazole_Mucosal | Imidazole mucosal | G01AF01 |
| AB_Imidazole_Topical | Imidazole topical | D06BX01 |
| AB_Imidazole_Systemic | Imidazole systemic | P01AB01, P01AB02 |
| AB_Lincosamide_Mucosal | Lincosamide mucosal | G01AA10 |
| AB_Lincosamide_Topical | Lincosamide topical | D10AF01 |
| AB_Lincosamide_Systemic | Lincosamide systemic | J01FF01 |
| AB_FQN_Topical | Fluoroquinolone topical | S01AE01, S03AA07 |
| AB_FQN_Systemic | Fluoroquinolone systemic | J01MA01, J01MA02, J01MA12 |
| AB_Antiparasitic | Antiparasitic | P01BA02, P01BB51, P01BC02, P02CA01 |
| AB_AntiTB | Anti-tuberculosis | J04AB02 |
| AB_Other_Topical | Other topical | D06AX01, D06AX09, S01AA13 |
| AB_Other_Systemic | Other systemic | J01XB01, J01XE01, J01XX0 |
| AB_PEN | Penicillin | J01CA04, J01CE02, J01CE08, J01CE20,  J01CR02 |
| AB_CEPH | Cephalosporin | J01DB01, J01DC02, J01DD04 |
| AB_Chloramphenicol_Topical | Chloramphenicol topical | D06AX02, S01AA01, S01AA32 |
| AB_BL_Any | Beta lactam | AB_CEPH, AB_PEN |
| AB_Any_Mucosal | Any mucosal antimicrobial agent | AB_Lincosamide_Mucosal, AB_MYC_Mucosal, AB_AG_Mucosal, AB_Imidazole_Mucosal |
| AB_Any_Topical | Any topical antimicrobial agent | AB_MYC_Topical, AB_AG_Topical, AB_Antiviral_Topical, AB_SULFA_Topical, AB_Chloamphenicol_Topical, AB_Imidazole_Topical, AB_Lincosamide_Topical, AB_FQN_Topical, AB_Other_Topical |
| AB_Any_Systemic | Any systemic antimicrobial agent | AB_MYC_Systemic, AB_AG_Systemic, AB_Antiviral_Systemic, AB_SULFA_Systemic, AB_MCD, AB_TTCN, AB_Imidazole_Systemic,  AB_FQN_Systemic, AB_Antiparasitic, AB_AntiTB, AB_Other_Systemic, AB_PEN, AB_CEPH, AB_BL_Any, AB_Lincosamide_Systemic |
| AB_Any | Any antimicrobial agent | AB_Any_Mucosal, AB_Any_Topical, AB_Any_Systemic |
