# Supplementary material for: Association Between Antenatal Antimicrobial Therapy and Autism Spectrum Disorder—A Nested Case-Control Study
Source: Front Psychiatry. 2021 Nov 19;12:771232. doi: 10.3389/fpsyt.2021.771232 (PMC8639519; doi:10.3389/fpsyt.2021.771232)
Supplement: Supplementary file 2 [file Table_2.DOCX]

**Supplementary Table S2** | Demographic and clinical characteristics of mothers stratified by ethnicity

| Variable | Jews  N=1914 | Bedouins  N=792 | P-value |
| --- | --- | --- | --- |
| **Demographic characteristics** | | | |
| **Age [mean years(±SD)]** | **30.08 (± 5.1)** | **26.72 (± 5.8)** | **<0.01** |
| **Socioeconomic status [median (IQR)]** | **9 (6-12)** | **6 (0-3)** | **<0.01** |
| **Chronic medical conditions** | | |  |
| Obesity [N(%)] | 122 (6.4%) | 57 (7.2%) | 0.46 |
| Any psychiatric [N(%)] | 38 (2%) | 13 (1.6%) | 0.67 |
| Depression | 27 (1.4%) | 9 (1.1%) | 0.71 |
| Other Psychiatric | 12 (0.6%) | 2 (0.3%) | 0.37 |
| Epilepsy [N(%)] | 16 (0.8%) | 7 (0.9%) | 1 |
| **Asthma [N(%)]** | **169 (8.8%)** | **30 (3.8%)** | **<0.01** |
| Atopic dermatitis [N(%)] | 78 (4.1%) | 31 (3.9%) | 0.95 |
| Diabetes mellitus [N(%)] | 74 (3.9%) | 24 (3%) | 0.36 |
| Any hypertension [N(%)] | 72 (3.8%) | 25 (3.2%) | 0.53 |
| **Thyroid [N(%)]** | **143 (7.5%)** | **29 (3.7%)** | **<0.01** |
| Hyperlipidemia [N(%)] | 17 (0.9%) | 4 (0.5%) | 0.43 |
| Rheumatic [N(%)] | 47 (2.5%) | 18 (2.3%) | 0.90 |
| **Chronic medications** | | | |
| Antiepileptic [N(%)] | 25 (1.3%) | 6 (0.8%) | 0.31 |
| **Psychiatric any [N(%)]** | **63 (3.3%)** | **11 (1.4%)** | **0.01** |
| **SSRI** | **33 (1.7%)** | **5 (0.6%)** | **0.05** |
| Asthmatic [N(%)] | 76 (4%) | 31 (3.9%) | 1 |
| Inhaled adrenergic [N(%)] | 49 (2.6%) | 25 (3.2%) | 0.45 |
| Antihyperglycemic [N(%)] | 14 (0.7%) | 10 (1.3%) | 0.26 |
| Antihypertensive [N(%)] | 18 (0.9%) | 8 (1%) | 1 |
| **Antacids [N(%)]** | **139 (7.3%)** | **100 (12.7%)** | **<0.01** |
| Glucocorticosteroids [N(%)] | 52 (2.7%) | 23 (2.9%) | 0.87 |

Bold text indicates significant group differences at α<0.05
